# The New Porcine Epidemic Diarrhea Virus Outbreak May Mean That Existing Commercial Vaccines Are Not Enough to Fully Protect Against the Epidemic Strains

**DOI:** 10.3389/fvets.2021.697839

**Published:** 2021-07-05

**Authors:** Qi Gao, Zezhong Zheng, Heng Wang, Songqiang Yi, Guihong Zhang, Lang Gong

**Affiliations:** ^1^College of Veterinary Medicine, South China Agricultural University, Guangzhou, China; ^2^Guangdong Laboratory for Lingnan Modern Agriculture, Guangzhou, China; ^3^Key Laboratory of Zoonosis Prevention and Control of Guangdong Province, South China Agricultural University, Guangzhou, China; ^4^National Engineering Research Center for Breeding Swine Industry, South China Agricultural University, Guangzhou, China; ^5^Agricultural Technology Extension Center of Jiangxi Province, Nanchang, China

**Keywords:** cross protection, phylogenetic analysis, porcine epidemic diarrhea virus, strain variation, PEDV

## Abstract

**Background:** On October 30, 2020, piglets and sows in the farrowing house of a pig farm in Jiangxi showed clinical symptoms such as anorexia, watery diarrhea, and vomiting. Epidemiological test, clinical necropsy, and RT-PCR test were carried out on the pig farm for diagnosis. After comprehensive considerations, the disease was judged as porcine epidemic diarrhea virus infection.

**Results:** Thereafter, a series of comprehensive prevention and control measures such as emergency vaccination with autogenous vaccines were adopted. Half a month after inoculation with autogenous vaccines for the farm, the mortality rate of newborn piglets in the farrowing house began to decline, and production gradually returned to being stable. The second-generation sequencing analysis and phylogenetic analysis showed that the porcine epidemic diarrhea virus (PEDV) sequence obtained from the stool and small intestine samples of the diseased pigs on the farm was 97.8% homologous to the vaccine strain. At the same time, antibody testing found that the vaccinated pigs on the pig farm had satisfactory immune response.

**Conclusion:** This case indicated that the PEDV outbreak on the pig farm might aggravate owing to the strain being mutated and could escape the immune protection of the existing vaccine. This case has accumulated technical data for the clinical prevention and control of porcine epidemic diarrhea.

## Introduction

Porcine epidemic diarrhea is an acute and highly contagious intestinal infectious disease caused by porcine epidemic diarrhea virus infection. The disease is prevalent in the cold season and is transmitted through fecal–oral transmission. Its clinical symptoms include elevated body temperature, anorexia, vomiting, diarrhea, dehydration, etc., (1). Porcine epidemic diarrhea virus (PEDV) is a single-stranded RNA virus belonging to the genus *Coronavirus*, family *Coronaviridae*, with a genome length of 28 kb (2). The disease was first discovered in England and Belgium in 1971 (3) and successively discovered in China in the early 1980's (4). Since October 2010, a new variant strain of porcine epidemic diarrhea has appeared, which has been widespread in China and other major pig-raising countries in the world, and its prevalence in East Asia and North America is severe, causing large economic losses to the farms (5, 6). PEDV infection has become one of the main reasons for the high mortality of suckling piglets.

Vaccination is an important way to control infectious diseases. In 1994, Ma et al. (7) used the attenuated CV777 strain to prepare an inactivated vaccine with aluminum gel adjuvant. After immunizing piglets, the active and passive protection efficiency exceeded 85%. In 1995, the study team successfully developed a commercial transmissible gastroenteritis virus (TGEV) + PEDV bivalent vaccine. In 1998, Tong et al. confirmed that the CV777 strain obtained by continuous *in vitro* passage for 90 generations is suitable for the preparation of attenuated vaccines. In 1999, they successfully developed a PEDV + TGEV bivalent vaccine. The ratio of virion counts of TGEV to PEDV was 1:1. After immunization, the active and passive protection rates against PEDV were as high as 97.7 and 98%, respectively (8, 9). The PEDV vaccine strains in these two commercial vaccines were both classic CV777 strains. Before 2010, these two bivalent vaccines were widely used in China, effectively controlling the spread of PEDV and TGEV.

After the outbreak of PED in the United States in 2013, Collin et al. prepared an inactivated vaccine of the American isolate (G2 genotype) and proved that inactivated PEDV can induce sufficient humoral immunity level in immunized 4-week-old piglets (10). The PEDV strain used in the PEDV–TGEV and PoRV trivalent attenuated live vaccine produced by the Harbin Veterinary Research Institute subordinated to the Chinese Academy of Agricultural Sciences in early 2015 was still CV777. The PEDV strain in the bivalent vaccine marketed in November of the same year was ZJ08, which belongs to the G1b subtype (11). In December 2017, the PEDV bivalent inactivated vaccine and attenuated vaccine prepared by Prof. Xiao of Huazhong Agricultural University were successfully marketed. The vaccine strain was AJ1102, which was isolated in 2011, and belongs to the G2b type (12).

In the winter of 2020, a new wave of PEDV epidemic swept across China. The question is, why was there a large-scale outbreak of PEDV under the conditions that biosafety control was so strict under a non-plague background, and whether new strains have emerged. Here, we report a clinical case of PEDV in Jiangxi, which indicates that China's existing PED variant vaccines may not fully protect against the PEDV epidemic strain.

## Case Presentation

### Case Farm Characteristics

Recently, a case of PEDV infection occurred in a pig farm in Jiangxi. There were 8,082 sows on hand in the mating house of the pig farm, which were divided into 10 groups, with 800 pigs in each group. There were 902 sows in the farrowing house, with 80 farrowing beds as a unit and five units constituting a building. Before the onset of the disease, the farm recorded 25 weaned piglets per sow per year, and the loss of farrowing house was 8%. Loss from conservation and fattening was <6%. The pig farm regularly tested for enteric pathogens, and TGEV/PDCOV and PEDV had not occurred in the last 2 years. There had often been sporadic cases of porcine reproductive and respiratory syndrome (PRRS) on the pig farm.

Routinely used immunization regimens include vaccination of sows with live PRRS vaccine, swine fever vaccine, live pseudorabies vaccine, foot-and-mouth disease O+A vaccine, Japanese encephalitis vaccine, and parvovirus vaccine. Sows were immunized with a live PED vaccine and an inactivated vaccine at 30 days before delivery and 14 days before delivery, respectively.

### Clinical Presentation and Interventions

**Figure 1:**
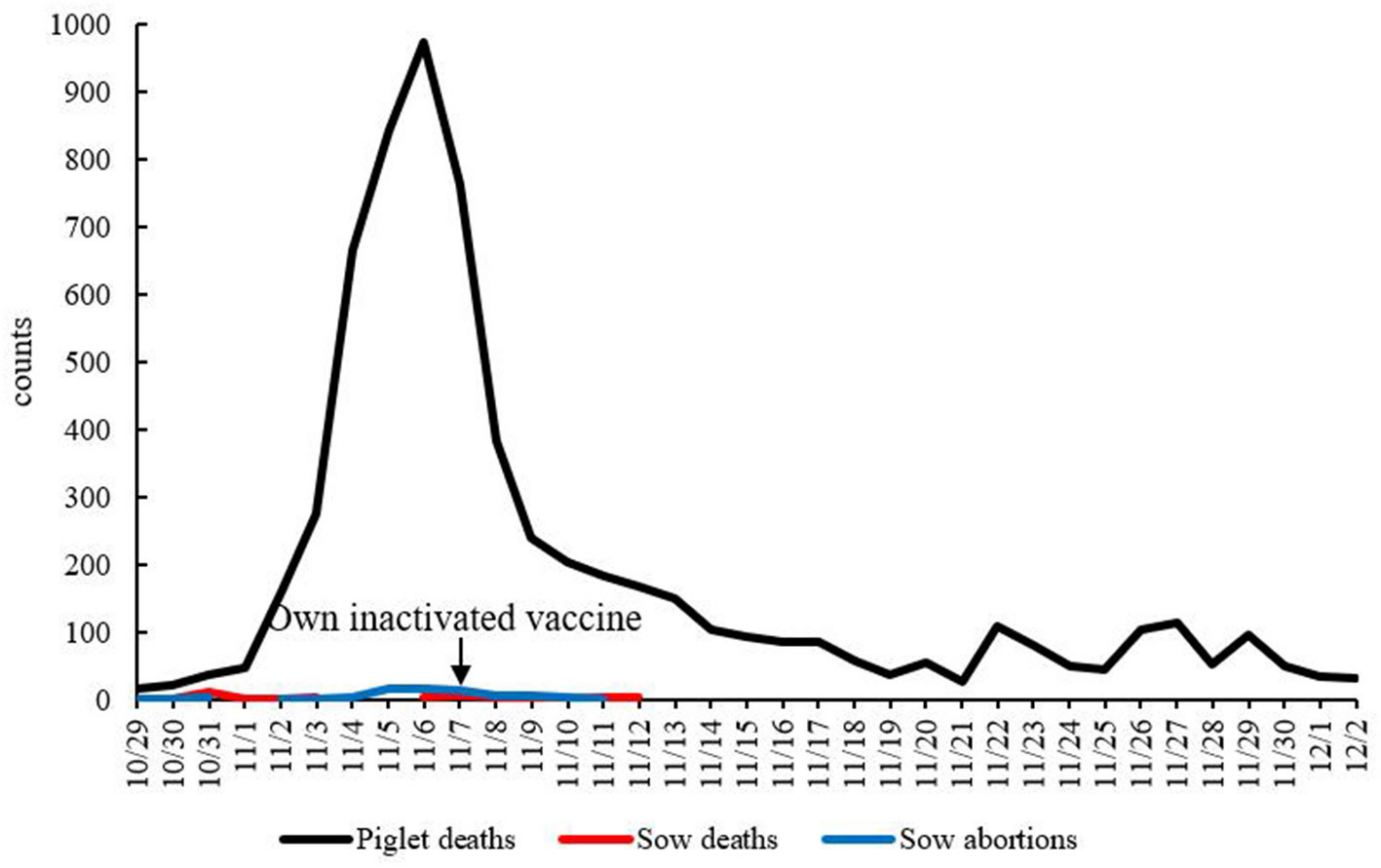
Time–mortality dynamic curve. The *x*-axis shows time, and the *y*-axis shows the number of died pigs. The arrow is the time for the farm to inject its own inactivated vaccine.

On October 28, 2020, at first, 80% of the sows in a farrowing unit developed depression, fatigue, and anorexia. Three days later, they developed mushy persistent diarrhea. The body temperature was normal or slightly higher, and they showed anorexia and had fully recovered about 7 days after onset. Piglets presented with epidemic diseases with watery diarrhea and vomiting as the main clinical symptoms. Vomiting usually occurred after eating or feeding on breast milk. The severity of symptoms varied with age. The younger the age, the more severe the symptoms. In a period of 1–3 days after newborn piglets within 7 days of age developed diarrhea, they were severely dehydrated and died, with a mortality rate of 90 to 100%. Diarrhea occurred successively in sows in other units 5 days later. After on-site anatomy and laboratory testing of feces and tissues, PEDV was determined to be the pathogen underlying this epidemic. Therefore, on October 31, the staff closed the pigsty where diarrhea occurred, treated the sows and piglets with amoxicillin- or lincomycin-containing drinking water for healthcare, and disinfected the pigsty with Denaishuang dry powder and kept it dry on November 7; intramuscular injection of autogenous vaccine was administered on November 11. The main ingredients of Denaishuang dry powder are essential oils, plant extracts, yucca products, seaweed products, and kaolin. Denaishuang dry powder plays a role in disinfecting the pig house and keeping it dry. The farrowing house was disinfected, the pigsty was dried and decontaminated with hot water at 60°C, the sick pigs were given salt solution for replenishment, and the farrowing house was cleaned, disinfected, and dried; on November 13, weaned piglets with secondary infection were treated with antibiotics. On November 16th, the sow swinery of the mating house was restored, the personnel were released from lockdown, and the empty swinery units were cleaned and disinfected. After adopting a series of comprehensive prevention and control measures such as emergency vaccination with autogenous vaccine, the mortality rate of newborn piglets on the farrowing room of this farm dropped significantly at half a month after vaccination, and production gradually returned to stable (Figure 1).

In general, there were four units with the most severe clinical symptoms of PED on this farm, and the mortality rate of suckling piglets was 40%. On this farm, a total of 85 sows died and 187 litters had abortion, with an incidence rate of 30%, and a total of 6,455 piglets died, with an incidence rate of 70%. At the current price, the net profit per pig was 300 US dollars, and the direct loss was nearly two million US dollars. Some weaned piglets also experienced diarrhea, weight loss, and growth retardation. The total loss from piglet feeding was 12%. In the fattening house, there was no sign of PED.

Epidemiological investigations revealed that at half a month before the outbreak of PED, PEDV broke out in another pig farm 800 m away from the farm.

### Laboratory Findings

**Figure 2:**
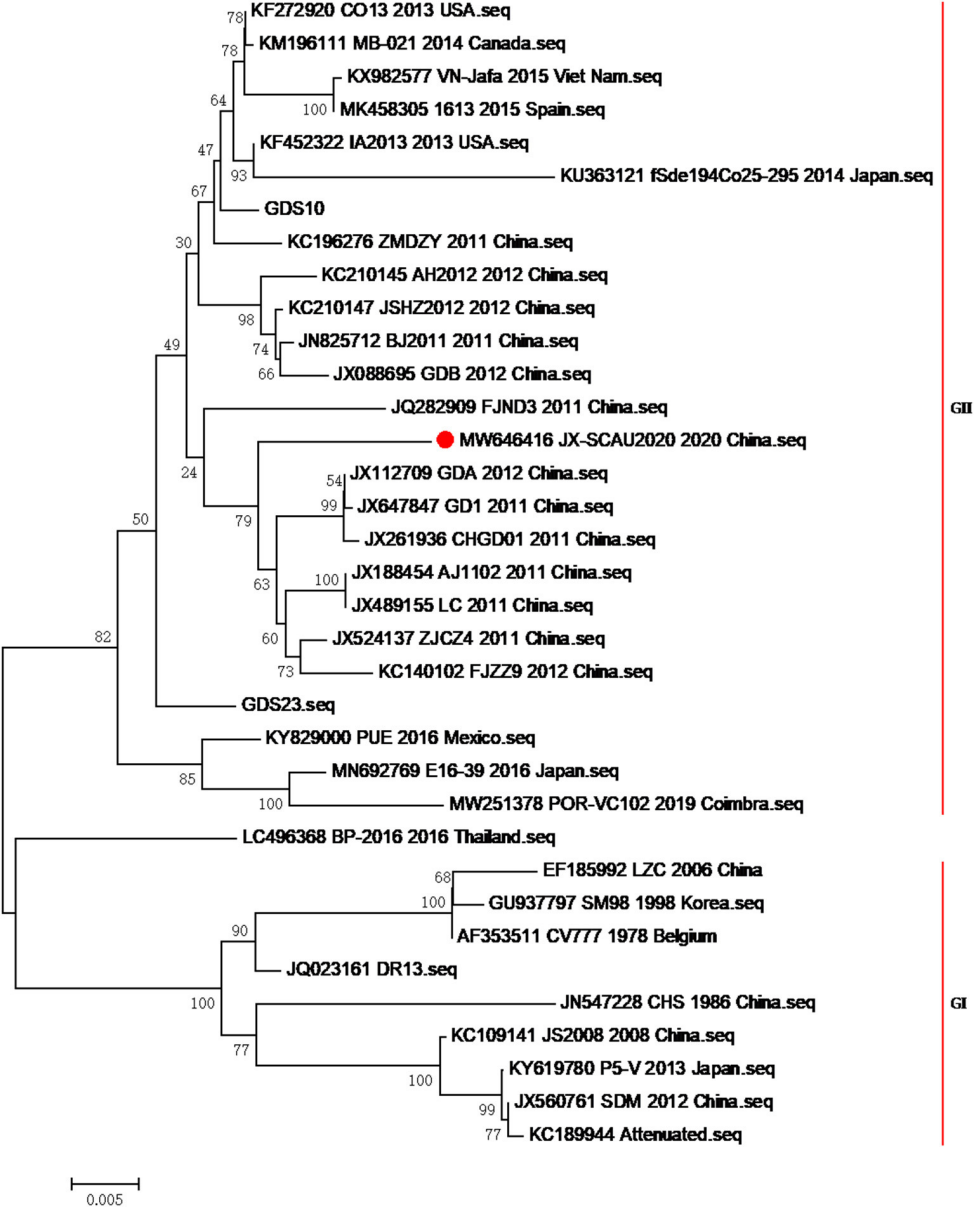
Phylogenetic trees of isolates and a representative virus based on the S gene sequence. The tree was performed by neighbor-joining method using MEGA with 1,000 bootstrap replicates. The GenBank accession numbers, strains, years, and places of isolation are shown on the right in order, and the genogroups of the virus are shown on the right of strains.

After preliminary confirmation of the PEDV pathogen by the pig farm laboratory, fecal samples, milk, and serum of 20 sows, as well as the feces and intestines of three infected piglets, were sent to South China Agriculture University for sequence analysis and serological testing. In order to reveal the molecular characteristics of the virus and determine the relationship among the current epidemic strains in China and other subtype strains, the bootstrap method of MEGA6.0 software was used to analyze the evolutionary tree of domestic isolates and reference strains from different sources at different times, which indicated that the strain formed a new and unique branch in the PEDV genotype II (Figure 2), which had the closest genetic distance to the genotype I strain. The base sequence of the S1 gene has been uploaded to NCBI, and the GenBank number is MW646416. The S1 gene homology comparative analysis of this strain showed that it had 98.1–100% homology with 59 epidemic strains uploaded by NCBI in the past year, 91.8% homology with the CV777 strain, 97.8% homology with the AJ1102 vaccine strain, and 97.3–98.1% homology with the 89 strains prevalent in 2014–2015 measured by our laboratory (Table 1), indicating that the PEDV of the current outbreak had mutated. Please see Supplementary Figure 1 for specific similarities. The similarity with the 2020 strain was also poorer by 1–2% compared with the 2014–2015 strain, indicating that PEDV had continuously mutated.

**Table 1:** Comparison of S1 gene homology between PEDV epidemic strain and vaccine strain in 2020.

**Strain comparison**	**S1 homology**
59 epidemic strains in 2020	98.1–100%
CV777 strain	90.3–92.1%
AJ1102 vaccine strain	96.8–97.9%
89 strains from 2014 to 2015	97.3–98.1%

To evaluate the immunizing effect of the PED vaccine in this farm, 20 diseased sows in farrowing season were randomly selected before the immunization with autogenous inactivated vaccines and numbered 1–20. Colostrum, feces, and serum were collected, respectively to test the levels of PED antigen and IgA antibody in the colostrum, as well as the levels of PED antigen in feces and specific IgG antibody in serum. The results showed that the pigs had a good immune response after immunization with the PED vaccine, with high levels of IgA and IgG antibodies. However, for the three sows numbered 8, 11, and 20, despite the high levels of IgA in milk and serum IgG antibodies, there were still detectable PED antigens (Table 2). PED antigens were detected in the feces of more sows, indicating that the antibody levels produced by the existing commercial vaccines cannot completely neutralize the new epidemic strain of PEDV.

**Table 2:** Analysis of antibody and virus shedding of infected sows.

**No**.	**Antigen**		**Antibody**	
	**Colostrum**	**Feces**	**Milk IgA**	**Serum IgG**
1		36.77	1.225	1.532
2		–	2.013	1.428
3		–	1.934	1.62
4		33.8	0.463	1.219
5		34.71	0.488	0.893
6		–	0.887	1.117
7		34.27	0.382	0.681
8	37.11	–	0.585	0.788
9		–	1.542	0.889
10		34.5	1.368	1.213
11	34.49	–	0.906	0.982
12		35.65	0.627	0.4136
13		–	1.327	1.348
14		–	2.323	1.787
15		–	0.185	0.685
16		–	2.086	1.782
17		–	0.041	0.182
18		32.12	0.862	0.464
19		–	0.808	0.686
20	38.21	35.32	0.332	0.514

To evaluate the fecal virus shedding of sows during the disease course, we conducted a 2-week monitoring of virus shedding on seven diseased sows. The results showed that the affected sows had intermittent virus shedding within 2 weeks after delivery. Some of the sows shed a great load of virus due to individual differences (Table 3).

**Table 3:** Monitoring results of continuous virus shedding of diseased sows.

**No**.	**11.3**	**11.4**	**11.5**	**11.6**	**11.7**	**11.8**	**11.9**	**11.10**	**11.11**	**11.12**	**11.13**
1	–	–	37.74	–	36.26	36.44	–	36.12	–	38.34	38.06
2	–	35.97	–	34.49	36.03	–	36.17	37.37	**30.66**	35.58	–
3	–	36.99	–	–	–	33.78	–	39.64	36.41	36.94	–
4	–	–	–	–	–	39.16	35.03	34.37	36.68	–	–
5	–	35.24	36.72	–	–	–	36.93	33.49	33.8	–	–
6	–	36.13	35.71	**30.21**	36.03	–	–	32.08	–	–	–
7	–	–	–	**29.87**	–	–	–	37.43	37.1	38.25	–

*Bold values indicate the copies of PEDV is the highest and the amount of pigs excreted is the most*.

## Discussion and Conclusions

In recent years, PEDV has repeatedly broken out in China and has become one of the deadliest pathogens infecting pig herds. Existing measures such as immunization, biosafety, and feeding with tissues from diseased animals cannot prevent its spread (13, 14). Although, vaccines prepared with G1a subtype strains have been developed and promoted for decades, it is precisely because of long-term use that its shortcomings are exposed. The amino acid mutation rate of the immunogen S gene of epidemic strains and vaccine strains has reached as high as 10% (15). The rapid development and promotion of new vaccines based on G2b subtype antigens have effectively prevented the spread of PEDV in China in the past 10 years. At present, all the genotypes of PEDV exist in China.

Owing to the pressure of herd immunity, the S gene of the coronavirus has frequently mutated, and changes in some amino acids have caused antigenic drift and escape from the protection of existing vaccines (16). Therefore, a periodic update of coronavirus vaccine strains becomes urgent. However, even the latest PED variant vaccine strain was isolated in 2011. For example, the antigenic difference has been confirmed in the virus neutralization experiment between the epidemic strain and vaccine strain of bovine coronavirus (17). Neutralization of the virus with hyperimmune serum showed that there is only one serotype of PEDV. However, an increasing number of field surveys show that a mismatch between virus sequence analysis and serum neutralization test indicates a big difference between the antigenicity of PEDV epidemic strain and the vaccine strain. To date, the attenuated vaccine of the mutant strain continues to play a partial protective role (18). However, an increasing number of pig farms, which have undergone normal immunization procedures, continue to have outbreaks of PEDV, and the isolated strains have confirmed it to be a highly pathogenic epidemic strain.

Following the occurrence of PED in the pig farm, the outbreak has spread fast and has incurred huge losses; thus, a vaccine strain that matches that of the epidemic in the pig farm must be selected for the immunization program. At present, there are only few PED vaccine strains available in China, and pig farms will most probably use autogenous inactivated vaccines for emergency immunization. Inactivated vaccines mainly act through humoral immunity and can produce a large amount of IgA. IgA antibodies are considered the most important protective antibodies in pig colostrum and provide a protective effect for piglets (19). Our research results show that although the vaccine could produce high titers of IgA antibody, some pigs still shed virus in the milk. This shows that the existing vaccine may not completely neutralize the PEDV epidemic strain. PEDV is mainly transmitted through feces and mouth, and the viral load carried in feces is very high. Data show that 1 g of feces dissolved in 100 L of water is highly infectious to pigs (20). Our continuous monitoring of the fecal virus shedding of diseased sows found that they could intermittently shed virus for more than 2 weeks; thus, it is particularly important to strengthen disinfection and adopt biosafety measures. From the perspective of the hazards of PEDV, before 2015, PEDV caused fulminant epidemics nationwide. Since 2016, as China has gradually gained a clear understanding of the prevention and control of PED, acute fulminant epidemics have decreased significantly. It is worth noting that the pathogenicity of highly pathogenic strains has not decreased.

To summarize, PEDV outbreaks on large-scale pig farms occurred regularly in the second half of 2020. The protective effects of inactivated and attenuated live PEDV vaccines available on the market are not satisfactory. The disease may develop despite elevated antibody titers following immunization with vaccines, which indicates that the existing vaccines may not provide adequate immune protection against epidemic strains. This may be related to the overall health of the pig herd and weak cross-protection between strains. The farm described in this report, where the infection occurred, could finally control the epidemic through immunization with autogenous inactivated vaccines, biosafety measures, and strict cleaning and disinfection. Therefore, the prevention and control of porcine epidemic diarrhea warrant comprehensive prevention and control measures. By appropriate selection of a suitable vaccine and effective management measures, ideal prevention and control may be achieved.

## Data Availability Statement

The datasets presented in this study can be found in online repositories. The names of the repository/repositories and accession number(s) can be found below: NCBI GenBank; accession no. MW646416.

## Author Contributions

LG analyzed the data and drafted the manuscript. QG and ZZ carried out most of the experiments. HW and SY participated in the study design. GZ conceived the study. All authors read and approved the final manuscript.

## Conflict of Interest

The authors declare that the research was conducted in the absence of any commercial or financial relationships that could be construed as a potential conflict of interest.

## Supplementary Material

Supplementary Figure 1 Percentages of nucleotide identity among 26 PEDV isolates based on S gene sequence.
