# Supplementary material for: The New Porcine Epidemic Diarrhea Virus Outbreak May Mean That Existing Commercial Vaccines Are Not Enough to Fully Protect Against the Epidemic Strains
Source: Front Vet Sci. 2021 Jul 5;8:697839. doi: 10.3389/fvets.2021.697839 (PMC8287018; doi:10.3389/fvets.2021.697839)
Supplement: Supplementary Figure 1 — Percentages of nucleotide identity among 26 PEDV isolates based on S gene sequence. [file Data_Sheet_1.pdf]

Percent Identity

	1	2	3	4	5	6	7	8	9	10	11	12	13	14	15	16	17	18	19	20	21	22	23	24	25	26		
1		99.4	98.9	98.8	98.8	98.5	98.8	98.5	98.8	97.9	98.0	98.2	98.3	98.2	98.4	98.3	98.3	91.4	91.6	92.0	92.9	91.5	91.5	92.2	91.7	97.3	1	KF452322_IA2013_2013_USA.seq
2	0.6		99.5	99.0	99.3	99.0	99.2	99.0	99.3	98.2	98.5	98.6	98.6	98.5	98.9	98.7	98.7	90.9	91.1	91.5	92.5	91.0	91.0	91.7	91.2	97.6	2	KF272920_CO13_2013_USA.seq
3	1.0	0.5		98.8	99.0	98.7	98.9	98.7	99.0	97.9	98.3	98.4	98.4	98.3	98.7	98.5	98.5	90.7	90.8	91.2	92.3	90.8	90.8	91.5	90.9	97.4	3	GDS10.seq
4	1.3	1.0	1.2		99.5	99.4	99.5	98.3	98.6	98.0	98.2	98.3	98.3	98.3	98.7	98.4	98.4	91.1	91.2	91.7	92.6	91.2	91.1	91.8	91.2	97.3	4	KC210145_AH2012_2012_China.seq
5	1.2	0.8	1.0	0.5		99.7	99.9	98.7	98.9	98.0	98.3	98.7	98.7	98.6	98.9	98.8	98.8	91.3	91.4	91.8	92.9	91.4	91.4	92.1	91.5	97.6	5	KC210147_JSHZ2012_2012_China.seq
6	1.5	1.0	1.2	0.6	0.3		99.7	98.3	98.8	97.8	98.1	98.4	98.5	98.4	98.8	98.5	98.5	91.0	91.2	91.6	92.5	91.1	91.1	91.8	91.2	97.3	6	JX088695_GDB_2012_China.seq
7	1.3	0.8	1.0	0.5	0.1	0.3		98.5	98.9	98.0	98.3	98.6	98.7	98.6	98.8	98.8	98.8	91.2	91.3	91.7	92.7	91.3	91.3	92.0	91.5	97.5	7	JN825712_BJ2011_2011_China.seq
8	1.6	1.0	1.2	1.7	1.4	1.7	1.5		98.8	97.9	97.7	97.9	98.0	97.9	98.3	98.0	98.0	91.1	91.2	91.5	92.7	91.3	91.1	91.8	91.3	97.3	8	GDS23.seq
9	1.2	0.8	0.9	1.4	1.1	1.3	1.1	1.3		97.9	98.1	98.3	98.3	98.2	98.5	98.4	98.4	90.8	90.9	91.3	92.4	91.0	90.9	91.6	91.1	97.3	9	KC196276_ZMDZY_2011_China.seq
10	2.2	1.8	2.0	2.0	2.0	2.3	2.1	2.2	2.2		97.4	98.0	98.1	98.0	97.8	97.8	97.8	90.8	90.9	91.4	92.4	91.2	91.0	91.7	91.2	97.3	10	JQ282909_FJND3_2011_China.seq
11	2.0	1.6	1.7	1.8	1.7	1.9	1.7	2.3	2.0	2.7		98.6	98.6	98.5	99.2	98.8	98.8	90.8	90.9	91.2	92.2	90.9	90.8	91.5	90.9	97.6	11	KC140102_FJZZ9_2012_China.seq
12	1.8	1.4	1.6	1.7	1.4	1.6	1.4	2.1	1.8	2.0	1.4		100.0	99.9	99.2	99.2	99.2	91.0	91.2	91.6	92.6	91.1	91.2	91.9	91.4	98.1	12	JX647847_GD1_2011_China.seq
13	1.8	1.4	1.5	1.7	1.3	1.6	1.4	2.1	1.7	2.0	1.4	0.0		99.9	99.3	99.3	99.3	91.1	91.2	91.6	92.6	91.2	91.2	91.9	91.4	98.2	13	JX112709_GDA_2012_China.seq
14	1.9	1.5	1.6	1.8	1.4	1.7	1.4	2.2	1.8	2.0	1.5	0.1	0.1		99.2	99.2	99.2	91.1	91.2	91.6	92.6	91.2	91.2	91.9	91.4	98.1	14	JX261936_CHGD01_2011_China.seq
15	1.6	1.1	1.3	1.4	1.1	1.3	1.2	1.7	1.5	2.2	0.8	0.8	0.8	0.8		99.4	99.4	91.1	91.2	91.7	92.5	91.3	91.2	91.9	91.3	97.9	15	JX524137_ZJCZ4_2011_China.seq
16	1.8	1.3	1.4	1.6	1.2	1.5	1.2	2.0	1.7	2.2	1.2	0.8	0.8	0.8	0.6		100.0	90.9	91.1	91.5	92.4	91.0	91.2	91.9	91.3	97.8	16	JX489155_LC_2011_China.seq
17	1.8	1.3	1.4	1.6	1.2	1.5	1.2	2.0	1.7	2.2	1.2	0.8	0.8	0.8	0.6	0.0		90.9	91.1	91.5	92.4	91.0	91.2	91.9	91.3	97.8	17	JX188454_AJ1102_2011_China.seq
18	8.2	8.7	8.9	8.5	8.4	8.6	8.5	8.6	8.9	8.9	8.9	8.6	8.6	8.6	8.5	8.7	8.7		99.8	99.5	98.0	96.1	95.7	96.3	95.7	90.7	18	KC189944_Attenuated.seq
19	8.0	8.6	8.8	8.4	8.2	8.5	8.4	8.4	8.8	8.7	8.8	8.5	8.4	8.4	8.4	8.6	8.6	0.2		99.5	98.0	96.1	95.7	96.3	95.7	90.8	19	JX560761_SDM_2012_China.seq
20	7.5	8.1	8.3	7.9	7.7	8.0	7.9	8.1	8.3	8.3	8.4	8.0	8.0	8.0	7.9	8.1	8.1	0.5	0.5		98.2	96.3	95.8	96.5	95.9	91.2	20	KC109141_JS2008_2008_China.seq
21	6.6	7.1	7.2	7.0	6.7	7.1	6.8	6.9	7.2	7.2	7.5	7.0	6.9	6.9	7.1	7.2	7.2	2.0	1.9	1.7		97.1	97.5	98.2	97.6	92.2	21	JQ023161_DR13.seq
22	8.2	8.8	9.0	8.5	8.4	8.7	8.5	8.5	8.8	8.6	8.9	8.7	8.6	8.6	8.5	8.8	8.8	4.0	3.9	3.8	3.1		95.3	96.1	95.5	90.8	22	JN547228_CHS_1986_China.seq
23	7.7	8.2	8.4	8.2	7.8	8.2	7.9	8.2	8.3	8.3	8.5	8.0	8.0	8.0	8.1	8.1	8.1	3.9	3.9	3.7	2.0	4.3		99.3	98.7	91.1	23	GU937797_SM98_1998_Korea.seq
24	7.4	8.0	8.2	7.9	7.6	7.9	7.6	7.9	8.1	8.0	8.2	7.8	7.7	7.7	7.8	7.8	7.8	3.7	3.7	3.5	1.8	4.1	0.2		99.4	91.8	24	AF353511_CV777_1978_Belgium.seq
25	8.0	8.6	8.8	8.5	8.2	8.5	8.2	8.5	8.7	8.6	8.8	8.4	8.3	8.3	8.4	8.4	8.4	4.3	4.3	4.1	2.4	4.7	0.8	0.6		91.2	25	EF185992_LZC_2006_China.seq
26	2.7	2.4	2.5	2.7	2.4	2.7	2.5	2.7	2.7	2.7	2.4	1.8	1.8	1.9	2.0	2.1	2.1	8.9	8.8	8.3	7.3	8.9	8.1	7.8	8.5		26	JX-SCAU
	1	2	3	4	5	6	7	8	9	10	11	12	13	14	15	16	17	18	19	20	21	22	23	24	25	26		

Table 1. Immunization program of basic sows and boars

Stage	Types of vaccines	Dose	Immunization method
First week of January	PRRSV live vaccine	1 piece	Intramuscular injection
First week of February	SFV vaccine	1 piece	Intramuscular injection
First week of March	PRV live vaccine	1 piece	Intramuscular injection
Second week of March	FMDV O+A vaccine	2mL	Intramuscular injection
First week of May	PRRSV live vaccine	1 piece	Intramuscular injection
Second week of May	JEV vaccine	1 piece	Intramuscular injection
First week of June	SFV vaccine	1 piece	Intramuscular injection
First week of June	PRV live vaccine	1 piece	Intramuscular injection
First week of July	FMDV O+A vaccine	2mL	Intramuscular injection
First week of September	PRRSV live vaccine	1 piece	Intramuscular injection
Second week of September	PEDV live vaccine	1 piece	Intramuscular injection
Third week of September	PRV live vaccine	1 piece	Intramuscular injection
First week of October	PEDV inactivated vaccine	2mL	Intramuscular injection
Second week of October	SFV vaccine	1 piece	Intramuscular injection
First week of November	FMDV O+A vaccine	2mL	Intramuscular injection
First week of December	PRV live vaccine	1 piece	Intramuscular injection

Table 2. Immunization program of sow

Stage	Types of vaccines	Dose	Immunization method
30 days before delivery	PEDV inactivated vaccine	1 piece	Intramuscular injection
14 days before delivery	14 days old	2mL	Intramuscular injection
14 days after delivery	PPV vaccine	2mL	Intramuscular injection

Table 3. Immunization program of Delivery room

Stage	Types of vaccines	Dose	Immunization method
1 day old	PRV live vaccine	1 piece	Nose drops
14 days old	PRRSV live vaccine	0.5 piece	Intramuscular injection
21 days old	PCV/Mycoplasma vaccine	0.5mL/0.5mL	Intramuscular injection

Table 4. Immunization program of fit pig

Stage	Types of vaccines	Dose	Immunization method
42 days old	PRRSV/PCV/Mycoplasma vaccine	0.5 piece/0.5mL/0.5mL	Nose drops
49 days old	SFV vaccine	1 piece	Intramuscular injection
49 days old	PRV live vaccine	1 piece	Intramuscular injection
63 days old	FMDV O+A vaccine	2mL	Intramuscular injection
77 days old	PRV live vaccine	2mL	Intramuscular injection
91 days old	FMDV O+A vaccine	2mL	Intramuscular injection
130 days old	FMDV O+A vaccine	2mL	Depends on the situation

Table 5. Immunization program of reserve pig

Stage	Types of vaccines	Dose	Immunization method
42 days old	PRRSV/PCV/Mycoplasma vaccine	0.5 piece/0.5mL/0.5mL	Nose drops
49 days old	SFV vaccine	1 piece	Intramuscular injection
49 days old	PRV inactivated vaccine	1 piece	Intramuscular injection
63 days old	FMDV O+A vaccine	2mL	Intramuscular injection
77 days old	PRV live vaccine	2mL	Intramuscular injection
91 days old	FMDV O+A vaccine	2mL	Intramuscular injection
112 days old	PRRSV live vaccine	1 piece	Intramuscular injection
133 days old	PRRSV live vaccine	2mL	Intramuscular injection
140 days old	PRV live vaccine	1 piece	Intramuscular injection
147 days old	PEV/PPV vaccine	1 piece/2mL	Intramuscular injection
161 days old	PRV inactivated vaccine	2mL	Intramuscular injection
168 days old	SFV/FMDV vaccine	1 piece/2mL	Intramuscular injection
175 days old	PEDV live vaccine	1 piece	Intramuscular injection
182 days old	PEV/PPV vaccine	1 piece/2mL	Intramuscular injection
196 days old	PEDV inactivated vaccine	2mL	Intramuscular injection
